# Image and perception of physicians as barriers to inter-disciplinary cooperation? – the example of German occupational health physicians in the rehabilitation process: a qualitative study

**DOI:** 10.1186/s12913-018-3564-1

**Published:** 2018-10-11

**Authors:** Jan M. Stratil, Monika A. Rieger, Susanne Voelter-Mahlknecht

**Affiliations:** Institute of Occupational and Social Medicine and Health Services Research, University Hospital Tuebingen, Wilhelmstrasse 27, 72074 Tuebingen, Germany

**Keywords:** Occupational health physicians, Primary care physicians, General practitioners, Rehabilitation, Interface, Health services research, Social identity approach, Conflicts, Cooperation, Inter-disciplinary

## Abstract

**Background:**

In the German rehabilitation system, primary care physicians (PCPs), occupational health physicians (OPs), and rehabilitation physicians (RPs) fulfill different distinct functions and roles. While effective cooperation can improve outcomes of rehabilitation, the cooperation between these groups of stakeholders has been criticized as lacking or insufficient. This article proposes an approach to understand the low levels of cooperation by examining the role of group perception and group identity in intra-professional cooperation as a barrier to cooperation between physicians in different roles. Group perception was evaluated in terms of (1) negative views about another group of medical specialists and (2) differences between the perception of members and non-members of a medical specialty group. To examine this issue, we focused on the role of OPs in the German rehabilitation process.

**Methods:**

We implemented a qualitative study design with eight focus group discussions with PCPs, OPs, RPs, and patients (two focus group discussions per stakeholder group; 4–10 participants) and qualitative content analysis. We used the Social Identity Approach by Tajfel and Turner as a theoretical underpinning.

**Results:**

While all protagonists reported a positive perception of their own professional group, we found numerous negative perceptions about other groups, especially regarding OPs. Negative perceptions of OPs included 1) apparent conflict of interest between employer and employee, 2) lack of commitment to patient outcomes, 3) lack of useful specialized knowledge which could have a bearing on rehabilitation outcomes, and 4) distrust on the part of their patients. We also found divergent perceptions regarding roles, responsibilities, and capabilities among the specialist groups. Both negative and conflicting perceptions about roles were characterized as barriers to cooperation by study participants.

**Conclusion:**

This example of cooperation between RPs, OPs, and PCPs suggests that negative and diverging perceptions about an out-group could create barriers in intra-professional and inter-disciplinary cooperation between physicians. These perspectives might also be useful in explaining problems at intersections between different specialties. We suggest examining the inter-group dimension of perception-based barriers to cooperation in future interventions to overcome problems caused by intra-professional and inter-disciplinary conflicts in addition to other barriers (i.e. organizational hurdles).

**Electronic supplementary material:**

The online version of this article (10.1186/s12913-018-3564-1) contains supplementary material, which is available to authorized users.

## Background

The complex and highly segmented German health care system is based on the cooperation of numerous specialized stakeholders with various professional competencies, organizational responsibilities, and goals. It is essential that these stakeholders are linked effectively through intersections, at which information is transformed, translated, and provided to the recipient. Intersections are points of transition in complex social systems. At these intersections specific professional expertise and skill levels, organizational responsibilities as well as the reach provided services end, which creates the need of continuation and supplementation in a cooperative manner [1]. While segmented health care systems can facilitate higher quality of services through specialization, they come with the risk of malfunctioning intersections leading to information loss or discontinuation of care pathways.

In the German rehabilitation process of working persons, the most important intersections between medical protagonists are those between primary care physicians (PCPs), occupational health physicians (OPs), and rehabilitation physicians (RPs). The focus of this study is on OPs. Every company in Germany is obliged to employ or work together with an OP, whose functions regarding rehabilitation include screening employees, initiating or supporting the process of applying for rehabilitation, providing RPs with information about the workplace, as well as assessing, preparing, and discussing occupational reintegration. This includes providing work accommodations (e.g. standing desks, supporting devices) and determining the need for retraining and job rotation. No referral by other physicians is needed. The role of this stakeholder is explained in more detail in our study protocol [2].

Absent or insufficient cooperation and communication at these intersections have been criticized for many years [3, 4]; in particular an insufficient flow of information from and to OPs [5, 6]. Surveys from Germany and other European countries found a low intensity of communication and cooperation between OPs and RPs [7–13]. German OPs in particular felt excluded from the rehabilitation process [10–13]. Although not focused on rehabilitation, insufficient cooperation between OPs and PCPs was reported in a literature review from Germany [14], as well as surveys from Germany [15], France [16], and Italy [17, 18].

All medical protagonists agree that improvements are needed [7–10, 12, 15, 19–28]. Several interventional studies from Germany indicated that improved cooperation could be beneficial in improving the occupational health of patients [29–37]. Furthermore, international literature reviews have identified several promising interventions, which cover the areas for which OPs are responsible in Germany [38–46].

As part of a larger research project aimed at identifying barriers and solutions in the cooperation between OPs, PCPs, and RPs [2], previous studies based on the same data set have outlined various barriers to cooperation [47] and compiled suggestions of the participants for how the cooperation could be improved [48]. Although problems at the intersections of different medical disciplines clearly constitute an intergroup issue, the role of group-identity and group-perceptions is often ignored [49]. Therefore, the present study aims to provide a meta-level assessment to understand and explain barriers by focusing on the group dimension of cooperation.

## Methods

This study is based on an explorative, qualitative research design using the Social Identity Approach (SIA) by Tajfel and Turner [50–56] as the theoretical underpinning, eight focus group discussions (FGDs) for data collection, and qualitative content analysis [57] for data analysis. A more detailed outline of methods and research questions is provided in the study protocol [2] and the preceding publications [47, 48].

### Theoretical approach

Social Identity Theory (SIT) adresses intergroup behavior and perception, and was developed to describe social factors in the development of perceptions, which lead to prejudice and discrimination [50–53]. It states that social categories (i.e. being an OP) provide a definition of who one is in terms of being part of a self-concept. In a specific context (i.e. the health care system), one of those categories may gain relative importance within the self-concept and form the social identity of the person. Thereby, individuals categorize themselves as members of a social group and relate to other protagonists in the system based on this affiliation (seeing others as part of the in or out-group). A positive social identity is based extensively on favorable comparisons, in which the in-group is positively distinct from the relevant out-group. The identity content is thereby comprised of specific value-laden attributes and characteristics, which members of the in-group attribute to themselves and use to compare themselves with members of the out-group. As a result, the out-group perception may become stereotypical and pejorative, and inter-group interaction is based on group identities and may become competitive and discriminatory [51, 55, 56]. The process of group categorization is further elaborated in the Social Categorization Theory (SCT), which was developed based on SIT. In this theory, the social identity groups are cognitively represented in terms of prototypes. These prototypes are simplified, stereotypical, or idealized members of the in-group or out-group, which are based on an identity content attributed to the group. The prototypes may be actual members of the groups or non-existing idealized versions of them [51, 56]. SIT and SCT together form the concept of the Social Identity Approach (SIA).

### Study participants

Two FGDs with four to ten participants (on average seven) were conducted with each group of medical stakeholders (PCPs, PRs and OPs), as well as with rehabilitation patients. We aimed for maximal structural variation in the composition of our sample to represent the heterogeneity of the stakeholders involved [58]. The purposive sample is shown in Table 1. OPs were recruited via telephone from members of the Association of German Occupational and Company Physicians (Verband Deutscher Betriebs- und Werksärzte) in the region of Tuebingen/Stuttgart. PCPs were recruited via email from medical practices associated with the Division of General Practice/Family Medicine, University Hospital Tuebingen. RPs and patients were recruited in cooperation with the rehabilitation clinics Treatment Center Federsee (Therapiezentrum Federsee; specialization: orthopedics, oncology, and rheumatology) and the Huettenbuehl Clinic of the Rehabilitation Center Bad Duerrheim (Reha-Zentrum Bad Duerrheim, Klinik Huettenbuehl; specialization: psychosomatic and mental health).

**Table 1:** Characteristics of focus group participants

Physicians	Primary Care Physicians	Occupational Physicians	Rehabilitation Physicians	Patients	
Participants	*n* = 22	*n* = 9	*n* = 12	*n* = 15	Participants
Age Average [Median/(Range)]	57 / (40–67) Years	55 / (45–65) Years	48 / (34–58) Years	53 / (22–63) Years	Age [Median / (Range)]
Sex: nbr. Female	n = 9	n = 5	*n* = 6	*n* = 8	Sex: nbr- fem.
Work experience as physician	27 / (13–40) Years	29 / (12–39) Years	13 / (6–30) Years	One: *n* = 4 Two: *n* = 1 Three: *n* = 1	Previous rehabilitation therapies
Work experience in specialization [Median/(Range)]	21 / (7–33) Years	20 / (1–32) Years	11 / (3–31) Years		
Type of employment	Solo practice: *n* = 13 Group practice: *n* = 9	Employed at enterprise *n* = 1 Employed in occupational health service: n = 4 Freelance: *n* = 4	all employed at the rehabilitation clinics	21 days: *n* = 4 28 days: *n* = 3 35 days: *n* = 5 > 35 days: *n* = 3	Planned duration of rehabilitation (days)
Practice site	Urban: *n* = 2 Rural: *n* = 10 Mixed: *n* = 10	Urban: *n* = 5 Rural: *n* = 0 Mixed: *n* = 4	N.A.	Mental health *n* = 5 Musculoskeletal *n* = 5	Reason for rehabilitation
Practice size (patients per 3 months)	< 700: *n* = 2 700–1400: *n* = 14 > 1400: *n* = 5	Responsible for SME: *n* = 8	N.A.	Office work: *n* = 5 Industrial production: *n* = 3	Occupation
Rehabilitation applications [Median/(Range)]	35 / (5–50) per Year	N.A.	N.A.	Construction work: *n* = 1 Logistic sector: *n* = 1 Nursing care: *n* = 2 Pedagogue: *n* = 1 Cleaner: *n* = 1	
				Small or medium enterprises: *n* = 7	Type of employer
				Business has OP: *n* = 8 Patient knows OP: *n* = 7	Relationship to OP (responses by patients)
Setting of data collection	Meeting room in University Hospital Tübingen or in our institute in Tübingen	Meeting room in our institute in Tübingen & Conference room in Stuttgart	Meeting room in rehabilitation clinics	Meeting room in rehabilitation clinics	Setting of data collection

This table displays the characteristics of the focus group participants. The left side of the table displays the characteristics of the medical stakeholders (PCPs, OPs, RPs). The right side displays the characteristics of the patients

### Data collection

The semi-structured FGDs lasted between 85 and 99 min and were conducted between February and May 2015. The interviews were conducted by one of two female researchers working for the Institute of Occupational and Social Medicine and Health Services Research at the University Hospital Tübingen (an occupational safety engineer and an associate professor for occupational, social, and environmental medicine (author SVM)). Both have previous experience in conducting qualitative research and received theoretical training in our institute. We informed the participants prior to the FGD about the professional background of the interviewer and the aim of the research project. One of the interviewers was already acquainted with three OPs and one GP. A research assistant was present in one of the interviews. The FGD guide was developed by an interdisciplinary team of content and methods experts based on previous literature reviews [6, 14], and our main research questions were pilot-tested before use. It focused on the topics: (1) attitudes towards rehabilitation therapy (warm-up question), (2) the perceived role and function of OPs, GPs, and RPs in the rehabilitation process, (3) the informational need of patients and medical stakeholders, and (4) the perceived quality and intensity of cooperation and communication at the interfaces between the different groups. The full interview guide can be provided upon request.

### Data analysis

The discussions were digitally recorded on video and audio files. No field notes were taken. We used the methodological orientation of content analysis, the method of qualitative content analysis [57], and the software MAXQDA 11 (VERBI GmbH; Berlin, Germany) to assess the transcribed and pseudonymized interviews. The coding frame was developed inductively from the text while keeping the main research questions in mind. After we assumed saturation to be reached after three transcripts, we revised the coding frame and applied the categories deductively to all transcripts based on the research questions. To control for subjective blurring and to achieve intersubjective creditability, two to three persons applied the categories to the transcripts, in part independently and in close discussion [57]. The category system is provided in Additional file 1. We conducted a workshop for content validation in January 2015. Representatives of all participating groups were invited, and a total of 16 GPs and OPs participated.

## Results

First, we describe how the cooperation with OPs is perceived by the protagonists. Next, we outline the perception by OPs, PCPs, and RPs of their own professional group (in-group perspective), followed by a description of how the professional groups are perceived by other protagonists (out-group perspective). Then we focus on the process of group distinction, and finally we outline how the negative perceptions about other groups, as well as diverging perceptions between the groups relate to cooperation deficits.

### Cooperation with OPs in the rehabilitative health care system

OPs in both FGDs criticized being left out of the rehabilitation process or not being taken seriously. The perception of OPs being excluded from the rehabilitation process was confirmed by PCPs and RPs, and agreed with patient statements. They reported often not receiving information from the rehabilitation clinic. For example, OPs in one FGD stated that they sometimes learned about a rehabilitation therapy weeks or months after it was completed. An existing cooperation between PCPs and OPs was not reported by either side. According to OPs, RPs would seldom ask them to provide information; which was supported by statements from RPs. RPs in both FGD interviews argued that many of their patients either did not know the OP responsible for them or believed that they did not have one. Hence, RPs stated they were hesitant to cooperate with OPs due to the patients’ distrust. While arguing an improved cooperation between OPs and RPs was desirable in general, several RPs acknowledged rarely cooperating with OPs on occupational reintegration.

Excerpt from PCP-FGD-I: *Interviewer: “[When thinking about OPs], where do you see their position, their relevancy [in the rehabilitation process]?” PCP: “We don’t know, as we do not know what they are doing at the moment. In the past 18 years, I never been in touch with an OP. Other than sometimes patients coming to my practice […] and telling me that their OP had told them that their cholesterol or liver enzymes were elevated. But beyond that, I don’t hear anything. There really is no communication with OPs.”*

### Self-perception of medical protagonists

We found that all medical protagonists in the rehabilitation process tried to establish and maintain a positive social identity that was based on specific functions and characteristics that also made them essential for the rehabilitation process (e.g. OPs: profound knowledge of workplace). We found aspects of an idealized prototype of the own profession in all groups. For example, the image of the highly committed OP who actively guides his patients through the rehabilitation process like a lighthouse, or the RP who enables their patients to rejoin social and working life.

#### In-group perception of OPs

OPs in both FGDs perceived themselves as important protagonists, whose contribution was essential for a successful rehabilitation. According to them, OPs have the most profound knowledge about their patients’ occupation and about the other specialties involved in the process. Therefore, OPs would make ideal coordinators. OPs considered themselves to be experts concerning the interface of occupation and health, and perceived it as their field of responsibility whenever the rehabilitation process touched on this interface. The ideal image was the “highly committed OP” who is a “lighthouse” to the patients in the process. This role model OP actively screens for patients requiring rehabilitation, follows the patient’s progress, and is actively engaged in the interaction with RPs. However, the interviewees admitted that some of their colleagues might not share the same devotion to the issue of rehabilitation as they themselves felt. Although focused on the patient’s ability to work, OPs perceived themselves as advocates for the patient’s well-being. The patients’ ability to work was regarded as an essential part of well-being and necessary for participating in society. They argued that there was a strict division between them and the employers, and that patient-doctor-confidentiality was protected by a strict professional code of conduct and legal regulations. We found mixed perceptions among OPs concerning how patients perceive them. Some OPs stated they had a good and trustful relationship with their patients, and that OPs were perceived as mediators between the employer and the employee who sided with the patient. But the OPs understood that the patients’ perception of them was not uniformly positive.

Excerpt from OP-FGD-II: *“OP1: […] I do believe we provide a valuable contribution. Who really knows the work on site and can link health to occupation, and occupational burden to health?” OP2: “Yes, precisely” OP1: “That’s the OP!” OP2: “Yes, definitely” OP3: “He/She is the lighthouse!”*

#### In-group perception of PCPs

One central identity content of PCPs in the rehabilitation process was the role of patient advocate. PCPs constructed a self-image of physicians with strong and trustful doctor-patient-relationships. Many PCPs perceived their professional group as the protagonist with the most profound knowledge about their patients. PCPs described themselves as committed to improving their patients’ health and to defend it from harmful influences, such as arbitrary decision-making by the pension insurances or exposure to work-related hazards. PCPs portrayed themselves as having a high workload and little time to spare. In the rehabilitation process, they regarded themselves as door openers, enabling the right patients to receive the necessary rehabilitative treatment. For these reasons, PCPs described themselves as best-suited for the role of coordinator or case manager in the rehabilitation process.

#### In-group perception of RPs

The most prominent self-perception of RPs in our interviews was the ideal RP who enables the patients to rejoin social and working life. A highlighting characteristic was the focus on social well-being and occupational functioning. The RPs stated they had profound knowledge about the working environment and occupational burden of their patients. They viewed themselves as hard-working, highly committed to delivering good medical service, and to the interaction with other actors. However, they perceived their work as very stressful and felt confronted with high and often unjustified demands by their patients.

Excerpt from RP-FGD-I: “*What is health? Being able to live and work – according to Freud. And this is exactly what we [as RPs] do here”.*

### Perception of professional groups by others

#### Out-group perception of OPs

One prominent narrative among PCPs and rehabilitation patients about OPs was that they were not primarily working in the interest of their patients but were rather “henchmen” of the employers. One PCP stated that contacting OPs about occupational health risks would never lead to any change in the patient’s workplace situation, which he thought was due to OPs being corrupted by the employer. Rehabilitation patients were especially concerned regarding information being passed on by OPs to the employer. RPs reported these concerns to be prevalent among their patients. This aligns with a prominent narrative among RPs and PCPs regarding the doctor-patient relationship between OPs and patients, which was dominated by unfamiliarity and distrust. Most patients reported either not knowing their OP at all or having little to no contact. This was confirmed by RPs in their FGD. In contrast, two participants had a positive perception of OPs: They described the relationship as good and trustful. OPs were aware of these negative perceptions and strongly rejected them. They stated that OPs were sometimes perceived as opponents in the struggle for the patients’ health and well-being by PCPs. While most PCPs shared this negative perception, two PCPs held a more positive view about OPs and portrayed them as cooperative and willing to improve patient health. One PCP who reported having had positive experiences with OPs in the past stated that although friendly and willing to help, OPs were limited in their capacities by rules set by the employers. In both FGDs involving PCPs, OPs were described as having a weak work ethic, showing little commitment to the patient’s health and well-being, and having a well-paid job with short working hours. This was often mentioned in the context of OPs not being interested in cooperation, as PCPs had seldom experienced OPs trying to communicate with them. One OP stated that these kinds of perceptions were prevalent among PCPs.

RPs and PCPs perceived the role of OPs as unclear, optional, and RPs openly admitted that they considered the OP’s contribution rarely necessary. In some instances, these statements were followed by the acknowledgment that, in practice, cooperation with the OP was rare to nearly non-existent. RPs did not mention any disadvantages of low levels of cooperation nor benefits from improved cooperation. One RP did not know whether OPs were part of the occupational reintegration at all. When asked about the potential role of OPs in the rehabilitation process, PCPs in one FGD admitted to not knowing of any role OPs play or should play.

*Excerpt from PCP-FGD-I*: *“In most cases communication is established through our [PCP] initiative and is mostly a negative experience. […] [PCP gives an example of a patient he provided with a certificate of incapacity for a certain task]. This has never been successful, never! Instead, the patient returns to his workplace and the OP says: someone must do this job. This is why we get little joy from [cooperating] with them [OPs]. Because they just don’t care at all about our recommendations. […] They should be obliged to report why they can’t implement it. And they should be obligated to prove that they were not bought by the company and do not primarily work in the interest of the employer […].”*

#### Out-group perception of PCPs

OPs and RPs acknowledged the depth of the doctor-patient relationship between PCPs and their patients, and also shared the in-group perception of PCPs as being busy and having little spare time. One RP felt as if he was perceived as a disturbance when trying to communicate directly with PCPs. RPs and OPs both stated that PCPs had limited knowledge of their patient’s occupation and workplace. They assumed his was due to PCPs not assigning much value to this aspect of the patient’s life and not having insights into the working world. According to RPs, some PCPs did not properly understand the concept of “ability to work”. This was in part due to PCPs not reading the rehabilitation report, not being familiar with legal definitions, or not being sufficiently familiar with the patient’s occupation. OPs believed that some PCPs saw the employer as an antagonist in the struggle for improving patient health.

#### Out-group perception of RPs

PCPs described the RPs’ occupation as comfortable: RPs had short working hours and lower occupational stress than they had. Furthermore, RPs were described as not showing much commitment to cooperating. Both PCPs and OPs described RPs as being dishonest, as the content of rehabilitation reports were incongruent with the patient’s experiences (e.g. concerning results of therapy or number of procedures performed). PCPs accused RPs of glossing things over, as they had greater confidence in the patient’s side of the story. One PCP linked discrepancies in the reports to financial motives, since the reports are also sent to the insurance institutions. According to OPs, RPs lack objective knowledge about the patient’s occupation. They also said RPs depended on subjective and biased reports by the patients and therefore could not evaluate the occupational burden correctly. One OP argued that people training to become RPs tended not to be very dynamic or aimed to achieve ambitious goals. Later the perception was mentioned that due to recruiting problems many physicians working as RPs had an immigration background. Participants commented that this led to language problems and that these physicians tended to have a more superficial understanding of their profession.

Excerpt from OP-FGD-I: *“One has to ask oneself: who becomes an RP? […] Are these the ones who are most dynamic? Who want to achieve something? Or rather those who tell themselves: it is quite comfortable being in this position”*

### Group-based comparisons and distinctions

To a large degree, group distinctions were made by contrasting the goals and functions of the own group in the rehabilitation process with those of the other groups. In these comparisons, the participants highlighted how essential or important their own role was, or they created a distinction based on value-laden attributes.

For example: OPs characterized themselves as experts on occupation or the workplace, and contrasted PCPs and RPs based on their lacking knowledge or insights. The unique and essential role in the self-perception of OPs was a result of PCPs and RPs being dependent on subjective and biased reports by the patients and having little insight into the (real) working world, while, for example, OPs could objectively and correctly describe the actual work load. This core concept leads to the self-perception of OPs of being essential to the rehabilitation process, as only they can provide the insights into the work environment the other stakeholder lacks. RPs made a similar distinction to PCPs, also based on the limited knowledge of PCPs about the workplace.

Excerpt from OP-FGD-I: *OP1: “ [It is good that the PCP takes on the role of coordinator in the rehabilitation process]. But the [coordination] within the workplace, that is in good hands with us. Because, with a positive patient image, a positive scope of performance levels, and [knowledge of] the workplace requirements, we are much better-suited to evaluate what is possible and sensible.” OP2: “Yes, because the PCPs don’t have any insights. It would be presumptuous if they asserted they could do this.”*

An example for the distinctions through devaluing based on value-laden attributes is the clear, dichotomous distinction made by PCPs between themselves, the highly committed and diligent PCP working to help and protect their patients, and the well-paid OPs with short working hours and little commitment to the patient’s well-being. A similar distinction was made regarding RPs, who according to PCPs worked few hours and had low levels of occupational stress, while PCPs portrayed themselves as having a high workload and little time to spare.

*Excerpt from PCP-FGD-I: PCP1: “[…] I never felt the need or had particular interest [in cooperating with OPs]. Because we will talk to them over the phone for an eternity, and nothing comes out of it” […] PCP2: “Those OPs have an unsavory taste. […] It is a relaxed occupation: they start at 8 in the morning, are at home at 4 PM, and are well-paid for that by the company. They don’t have any responsibility; don’t need to spring into action during the night. [..] Maybe there is a little envy talking from our side.”*

Excerpt from PCP-FGD-I: *“The level of contact is close to nil. They sit around somewhere and have an easy job in my view. You can see that from their (lack of) availability in the morning at half past seven or in the afternoon after four PM. We have close to no points of contact”*

### Perception-based barriers to cooperation with OPs in the rehabilitation process reported by protagonists

In this section, we contextualize the diverging and negative group-based perceptions in the context of barriers to cooperation.

#### Henchmen of employer

A negative perception of OPs, in which they were put on a level with the employer, was often mentioned in the context of, or as a reason for low levels of cooperation. An OP, who had worked formerly as an internist in a hospital, stated that she had been skeptical about sharing information with OPs, due to the belief that OPs worked first in the interest of the employer. The same argument was put forward by PCPs and RPs to explain an unwillingness to provide sensitive information to OPs. Most PCPs who held negative views about OPs reported that they did not cooperate or communicate with OPs. RPs in both interviews stated that their patients’ negative perception of OPs was a barrier to cooperation between OPs and RPs. RPs argued that due to data privacy regulations, patients may decide who can receive personal information and would sometimes decline consent to providing information to the OP. The patients who reported to have a good relationship with their OP did not have reservations about them receiving information at the intersections. OPs were also aware of the negative attitudes among rehabilitation patients. They argued, this was due to insufficient knowledge among rehabilitation patients about the strict separation between the employer and the OP in the German health care system and the code of professional confidentiality.

*Excerpt from OP-FGD-I: “I am a doctor for internal medicine by training and have worked in the hospital for many years. Do you believe I would have taken an OP seriously? Not at all! […] What do they want? That is actually the employer! I won’t tell them anything!”*

*Excerpt from RP-FGD-I: “But to the company physicians, there’s hardly any contact, if any. And that has a lot to do, speaking from my own experience here, a lot to do with prejudices and fears [of the rehabilitants] that confidentiality will be neglected regarding their employers, etc.”*

#### OPs as optional protagonists

The in-group perspective of OPs and the out-group perspective by PCPs and RPs diverged considerably regarding roles in the rehabilitation process. OPs perceived themselves as having information and knowledge that was important and not available to PCPs and RPs, which made their contribution essential. By contrast, the role of the OP was considered optional or irrelevant by some PCPs and RPs. Some OPs had experienced that this perception was prevalent among PCPs and RPs. In one FGD with RPs, one RP stated that they were not aware of the potential function of OPs in the rehabilitation process. RPs argued that in practice, the delivery of workplace information and occupational reintegration was rarely needed. OPs in both interviews stated that cooperation deficits might be caused by insufficient knowledge of PCPs and RPs about OPs’ functions and potential role in the rehabilitation process.

#### Less dedicated & limited agency

The image of OPs as not working as hard as PCPs was quite prevalent among PCPs. PCPs argued that communicating with OPs was complicated by their short working hours, with long lunch breaks and leaving work early in the evening. These aspects of limited availability were associated with the perception of OPs as being less hardworking than PCPs. One PCP reported having little points of contact in the past, but that these had been positive. OPs were friendly and willing to help, but they were limited in their capacities by rules set by the employer.

*Excerpt from OP-FGD-I: “At the times when we have the time to call them, you cannot reach anyone, because it is lunch break again or after 7 PM”. “Actually, they [OPs] should be the ones responsible for trying to get in touch with us”*

## Discussion

In this article, we examined the role of group perception and group identity as barriers to cooperation between physicians in different roles. Perception was evaluated in terms of (1) negative views about another group of specialists, and (2) differences between the perceptions of one specialty group in comparison with others. In the context of the FGD on the rehabilitation process, our participants interacted with their peers based on their shared social category (being OPs, PCPs, or RPs). Thereby, the individuals categorized themselves as members of this particular social group and related to the other professional groups as members of the out-group rather than as individuals. This was highlighted by the extensive use of “we” and “they” in the statements. According SIA, the mere perception of belonging to two distinct groups can be sufficient to provoke intergroup competitive or discriminatory responses on the part of the in-group [48]. As predicted, the protagonists described a positive perception of their own professional group, while maintaining negative or prejudicial perceptions about other professional groups, especially regarding OPs. The group distinction and characterization were achieved through positive distinction, as well as through devaluing other protagonists. Several of these distinctions are shown in Table 2, which provides an overview of the ascriptions to the groups by its members (in-group) and those identifying with other groups (out-group). We also found divergent perceptions regarding roles, responsibilities, and capabilities among the specialist groups, and that these were put forward as reasons for non-cooperation or in the context of low levels of cooperation. The main concepts were (1) mistrust in OPs due to close ties to the employer, (2) low perceived need for and benefit from cooperation, and (3) OPs having low work ethics and limited agency to change anything.

**Table 2:** Reported self-perception of protagonists and perception of these groups by other medical protagonists

	OP	PCP	RP
In-Group perception	● Working in the interest of patients ● Profound knowledge of workplace; which others were lacking ● Experts on interface between occupation and health ● Well-suited to be coordinators in rehabilitation process ● Good relationship with their patients ● Role in rehabilitation process not known and adequately valued ● Important for successful rehabilitation process	● Hardworking ● Dedicated to patients ● Advocates for their patients ● Important for successful rehabilitation process ● Good and intensive relationship with patients	● Profound knowledge of patients workplace ● High workload and unjustified demand from patients ● Dedicated to cooperation with other protagonists ● Promoting patients’ physical health, social well-being, and occupational participation
Out-group perception	● Henchman of the employer ● Limited agency ● Not hardworking ● Not working in the interest of patients ● Patients don’t know them ● Role and function in rehabilitation process unclear ● Not interested in cooperation	● PCPs and OPs are competitors ● Not interested in cooperation	● Not interested in cooperation ● Insincere concerning reported rehabilitation outcomes ● Not interested in the patients’ health after end of rehabilitation ● Not hardworking ● Not very ambitious

This table displays the self-perception of protagonists (in-group perception) and perception of these groups by other medical protagonists (out-group perception) with regard to the rehabilitation of employees as reported by the medical stakeholders

### Identity and perception as roots of cooperation deficits

A negative image or stereotype of a group may be derived from real world events (e.g. negative experience of an individual OP). The problem can occur when, facilitated through group identity processes, the interaction with individual members of a group (e.g. OPs) are based on, or influenced by the stereotypical characteristics of the group, which are then ascribed to every individual (e.g. OPs are henchmen of the employers). This can pose a direct barrier to cooperation, for example when patients refuse to pass on information due to confidentiality concerns. Furthermore, they can shape how objective obstacles (e.g. structural barriers) limit cooperation. For example, PCPs not being able to be reached because of (objectively) different working hours, and PCPs not trying to contact OPs because they have the mental image of OPs having a weak work ethic and therefore assume OPs are difficult to reach. While it is not possible to distinguish these aspects based on the subjective accounts of our participants, we believe it is important to explore the extent to which structural barriers mask additional group perception-based hindering factors.

### Scientific literature on perception-based barriers to cooperation

Negative and/or sterotypical group perecptions have been reported in other studies: For example RPs being unaware of the OP’s role and function in rehabilitation [7, 8, 10, 13, 59]; patient unawareness of of the existence of OPs or their function, as well as patient mistrust of OPs as barriers to cooperation in the rehabilitation process [4, 7, 12, 27, 60–62]; or a lack of understanding of the OP’s role among physicians [19, 61, 63]. Several studies mention PCPs [14–18, 20, 59, 64, 65] or RPs [8, 9] mistrusting OPs (e.g. in terms of OPs not working in the interest of the patient or breaching confidentiality regulations).

Despite awareness about these barriers, the approach to understand them as perception-based barriers to cooperation at intersections resulting from inter-group processes has rarely been applied, either to examine interference in the cooperation of physicians in Germany, or in the rehabilitative health care setting. We only identified one Dutch study in which this concept was partially applied (without referencing SIA). The study found a correlation between trust of PCPs in OPs and the perceived relative status of the groups: PCPs who perceived their relative social status as higher reported having more professional trust in OPs [63].

While few studies examined the role of group perception and group identity in intra-professional cooperation between physicians, these issues were addressed in a number of studies focusing on inter-professional cooperation. For example, Kreindler et al. proposed SIA as a framework to understand inter-professional cooperation, i.e. between nursing staff and physicians [49].

### Facilitators and perquisites for successful cooperation

Several studies, including systematic reviews, identified facilitators and perquisites for successful inter-professional cooperation in the health care setting; especially concerning the cooperation of doctors and nurses [66–74]. These facilitators and perquisites included, i.e.: mutual trust [75–82], mutual respect [75–77, 79, 80, 83, 84], collegial partnerships [69, 75, 83, 85], understanding the practice of the other group’s profession [69, 75, 77, 86], awareness and valorization of other professionals’ contribution [76, 80, 85, 87], as well as perceived benefits of cooperation [85, 88, 89]. Conflicts were reported following a redistribution of power and functions of nurses, which was experienced as an erosion of roles by GPs [88–90]. A lack of clear understanding of the cooperation partner’s professional role and responsibility was mentioned as a barrier to cooperation and as promoting professional conflicts [66, 91, 92].

Despite a number of distinct differences, we believe that the intra-professional, inter-disciplinary conflicts in our study have some resemblance to inter-professional perception-based conflicts reported on in this study. We therefore argue that well-developed strategies to overcome barriers to inter-professional cooperation could also be useful in overcoming intra-professional cooperation deficits between different specialist groups of physicians.

### Approaches to overcome negative and prejudicial attitudes

One of these strategies identified by reviews on inter-professional is clearly-stated and shared goals as facilitators of successful cooperation in teams [66, 68, 69]. For the German rehabilitative health care system, an intervention to test this approach could focus on the development of shared goals in meetings with different disciplines (e.g. GP, RP, and OP), professions (e.g. physical therapists), and patients for the field as a whole or for circumscribed geographical regions.

Another approach to overcome inter-disciplinary group-based conflicts through facilitation of communication and relationship-building is joint educational programs [93]. These were suggested by participants in our study, as well as by the participants of other studies from Germany and the Netherlands [7, 9, 28, 65, 92]. One reason why participants in our study suggested introducing these programs was to overcome negative perceptions and attitudes through contact. The underlying theory is the contact hypothesis by Allport [94], which has been proven to effectively reduce prejudice and negative-attitudes [95]. While this approach was successfully applied in inter-professional cooperation, two studies on joint educational programs between OPs and PCPs did not show lasting positive impact [96, 97].

As a third approach, interventions building on the model to resolve intractable identity-based conflicts (IIC) could be used, which itself is based on the social-identity approach [98]. This model states that a de-escalation of identity-based conflicts drawing on negative perceptions about an out-group must go through stages of interaction. These stages are a (1) readiness and willingness to solve existing conflicts, (2) a decoupling of situation and conflict from inter-group identities, which may be achieved through the promotion of mindfulness about the situation. Thirdly, (3) establishing an secure identity within the subgroups can then be supported through promoting positive in-group distinctiveness, which does not draw on the devaluation of out-groups. Followed by a stage of (4) promoting cooperation around specific objectives while maintaining separate, distinct groups, and an (5) enduring intergroup harmony may be achieved through further promoting integrative goals and structures between the former conflicting identity groups [98]. We could not identify suitable interventional studies, which were based on the IIC-model.

### Strengths & Limitations

A strength of this study is the novelty of our approach, in which we looked at the low intensities of inter-disciplinary cooperation between GPs, OPs, and RPs in the German rehabilitation process by taking group perception and group-identity into account. Furthermore, we achieved high levels of heterogeneity in the composition of FGD-participants (e.g. working experience, disease profiles, company sizes) and included FGDs with patients.

Two limitations of our study result from the complicated recruitment process of OPs: a selective sample of OPs with a strong interest in the topic cannot be ruled out, and the composition of our FGDs deviated from the planned composition. As RPs and rehabilitants were recruited from two rehabilitation institutions, unwanted group effects cannot be excluded. Especially as studies indicate a pronounced heterogeneity of rehabilitation clinics regarding quality and interest in cooperation [99]. As the study was conducted by experts in the field of occupational health, biased responses due to social desirability are possible.

We conducted FGDs with homogenous professional groups to enable participants to have less constrained discussions and to allow them talk more freely about negative or possibly prejudicial attitudes. While we consider this approach successful, it would have strengthened the study if additional FGDs would have been conducted with mixed groups. This was partly achieved in the validation workshop held in January 2015, where both OPs and PCPs participated.

## Conclusion

Our explorative, qualitative study indicates that the deficits in cooperation between RPs, OPs, and PCPs in the German health care system could result from, or be influenced by perception- or group identity-based conflicts. A divergence between how group members (in-group) and other disciplines (out-group) perceive a group of specialists, as well as negative perceptions of one group could lead to conflicts and barriers to cooperation. We found these barriers especially pronounced regarding OPs. While this does not devalue the importance of other barriers to cooperation [47], we believe this group perspective could help understand and reduce these barriers. Lessons learned from interventions to improve inter-professional cooperation as circumscribed in this study might be useful to improve cooperation between physicians of different specialty groups.

Future quantitative research is required to assess the relative weight of the findings and to further explore our hypothesis. High-quality interventional studies incorporating models to overcome inter-group conflicts could advance this field.

## Additional file

Additional file 1: Category system used for coding the transcripts. (DOCX 73 kb)

## Abbreviations

FGD: Focus group discussion
IIC: Intractable identity-based conflicts
OPs: Occupational health physician
PCPs: Primary care physician
RPs: Rehabilitation physicians
SCT: Social categorization theory
SIA: Social identity approach
SIT: Social identity theory
