# Supplementary material for: Image and perception of physicians as barriers to inter-disciplinary cooperation? – the example of German occupational health physicians in the rehabilitation process: a qualitative study
Source: BMC Health Serv Res. 2018 Oct 11;18:769. doi: 10.1186/s12913-018-3564-1 (PMC6180505; doi:10.1186/s12913-018-3564-1)

# Additional file 1: Category system used for coding the transcripts

This figure describes the category system with the main categories, categories and subcategories.


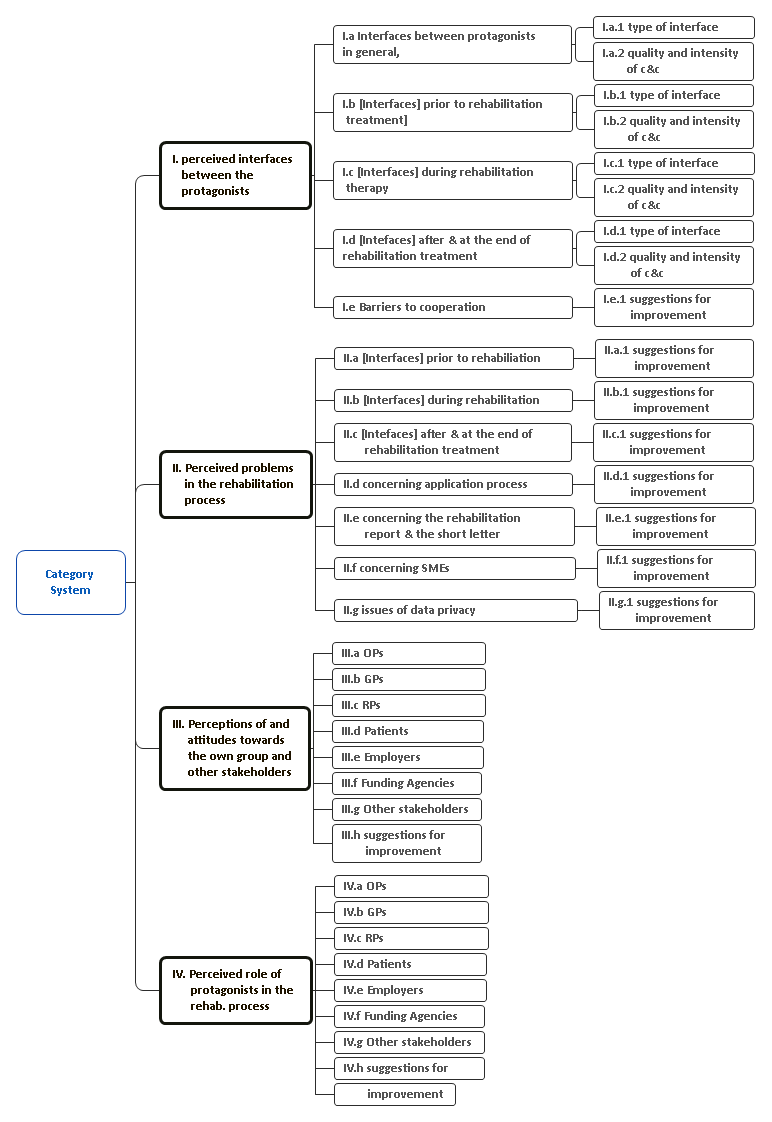

Supplement: Supplementary file 1 — Category system used for coding the transcripts. (DOCX 73 kb) [file 12913_2018_3564_MOESM1_ESM.docx]
